# Clinical Evaluation of Patients with Migraine Induced Stroke in Mashhad, Iran

**Published:** 2010

**Authors:** Kavian Ghandehari, Atena Sharifi, Zeynab Nikbin, Sahar Fadaei, Meysam Aghaei Meybodi, Mehdi Moshfegh, Mohammad Reza Hosseini, Mohammad Reza Gerami Sarabi, Parham Maarufi

**Affiliations:** 1 MD, Professor of Cerebrovascular Disease, Department of Neurology, School of Medicine, Mashhad University of Medical Sciences, Mashhad, Iran; 2 Md, Resident of Neurology, Department of Neurology, School of Medicine, Mashhad University of Medical Sciences, Mashhad, Iran; 3 Stroke Researcher, Mashhad University of Medical Sciences, Mashhad, Iran; 4 MD, Stroke Researcher, Tabriz University of Medical Sciences, Tabriz, Iran

**Keywords:** Migraine, Stroke, Migraine Induced Stroke

## Abstract

**BACKGROUND:**

Migraine Induced Stroke (MIS) is an important cause of brain infarction in the young people.

**METHODS:**

Consecutive patients with MIS admitted in Ghaem hospital, Mashhad during 2006–2010 enrolled a prospective clinical study. All of the patients suspected to MIS had brain MRI with a 0.5 Tesla generation, Philips NT Intra, Netherland. All of the MIS patients underwent a standard battery of diagnostic investigations for detecting etiology of stroke. Disability of MIS patients was detected based on the modified Rankin scale at 90 days post stroke.

**RESULTS:**

32 MIS patients (18 females, 14 males) with mean age 37.2 ± 3.8 years ranged 15–58 years were evaluated. Hypodense area of infarction corresponding to clinical manifestations was detected in MRI in 32% of our MIS patients. The mean disability score in our MIS patients was 1.09 ± 0.32, which is significantly lower than other stroke patients (z = 2.55, P = 0.007)

**CONCLUSION:**

MIS is an important cause of stroke in Persian young adults which have good prognosis.

## Introduction

Migraine Induced Stroke (MIS) or infarcts due to unusually severe hypoperfusion during aura, are rare and vastly overdiagnosed. They occur in patients with migraine with aura, during an attack of migraine with aura, with symptoms that are those of the aura with a documented infarct in the relevant area and in the absence of other causes at an extensive workup.^1^

The number of strokes attributed to migraine varies from 4% to 20%.^2^ Before diagnosing MIS it is important to exclude other co-existing conditions.

The overall lifetime prevalence of migraine is 10–16% and for the majority of patients with migraine who have a stroke, migraine is not the cause^2^. There are similar reports of symptomatic migraine occurring in young patients with carotid dissection and it is possible that many MIS in early series were carotid dissection.^2^ A meta-analysis of 11 case- control studies and 3 cohort studies revealed that the relative risk of migraine with aura for ischemic stroke is 2.27.^3^ A prospective cohort study of 27840 participants in the Women's Health Study determined that, compared with women lacking a migraine history, women who reported active migraine with aura had adjusted hazard ratios of 1.9 for ischemic stroke.^4^ The precise mechanism of migraine induced stroke is still; a matter of speculation, however inducing cerebral microcirculatory vasoconstriction, cortical spreading depression-related oligemia, intracerebral large vessels spasm and vascular endothelium related hypercoagulability were assumed as its mechanism.^5^ This is the first reported case series of MIS from Iran.

## Materials and Methods

Consecutive patients with MIS admitted in Ghaem hospital, Mashhad during 2006–2010 enrolled a prospective clinical study. All of the patients suspected to MIS had brain MRI with a 0.5 Tesla generation, Philips NT Intra, Netherland. A complete past medical history and neurologic examination was taken in all of the suspected MIS patients by a neurologist. MIS was detected based on below criteria: 1-The present attack in a patient with 1–2 migraine with aura is typical of previous attacks. 2- Either A or B is present. A: one or more aura symptoms persist for more than one week. B: one or more aura symptoms persist more than one hour and neuroimaging demonstrates infarction in relevant area. 3-Not attributed to another disorder. The diagnostic criteria is defined based on the first classification of international headache society.^6^ Although diagnostic criteria of MIS, based on the second edition of international headache society classification, necessitates that neuroimaging should demonstrate infarction in a relevant area in patients^7^, however we had a 0.5 Tesla generation of MRI in our hospital, which could easily lose detection of MIS. All of the MIS patients underwent a standard battery of diagnostic investigations for detecting etiology of stroke.^8^, ^9^ Etiology of MIS was determined based on the Asian Stroke Criteria.^10^ Disability of MIS patients was detected based on the Modified Rankin Scale (MRS) at 90 days post stroke^11^ and compared with a similar number of age and gender matched, randomly selected, ischemic stroke patients with other etiologies from our stroke registry. Follow up examinations were performed when possible, and when not, information about disability was gathered by telephone. Research project was approved by the ethics committee of Ghaem hospital. An informed consent signature was taken from the patients or his/her first degree relatives. Mann Whitney U test and independent T test served for statistical analysis.

## Results

32 MIS patients (18 females, 14 males) with mean age 37.2 ± 3.8 years ranged 15–58 years were evaluated. All of these cases had migraine with aura. Recent Oral contraceptive consumption was found in 22.2% of females with MIS. Hypodense area of infarction corresponding to clinical manifestations was detected in MRI in 32% of our MIS patients and other patients had normal brain MRI. The mean disability score in our MIS patients and also in age, gender matched, randomly selected other ischemic stroke patients was 1.09±0.32 and 3.12±0.83 at 90 days post event, respectively. Difference in mean disability score of these two groups of ischemic stroke patients was significant (z=2.55, P=0.007. Table 1 represents clinical characteristics of the studied 32 MIS patients.

**Table 1:** Clinical characteristics of 32 studied MIS patients.

Number	Gender	Age (y)	MRI findings	Trritorial involvement based on Clinical Exam	90 days disability score
1*	M	52	PICA, PCA, brainstem	Posterior Circulation	3
2	F	30	PICA	Posterior Circulation	2
3	F	46	Normal	Anterior Circulation	1
4	M	52	Normal	Ophthalmoplegic Migraine	1
5	M	44	Normal	Anterior Circulation	1
6	M	43	Normal	Anterior Circulation	1
7	F	41	MCA, Superior branch	Anterior Circulation	1
8	F	58	Normal	Anterior Circulation	0
9	M	15	Normal	Anterior Circulation	1
10	M	30	Normal	Anterior Circulation	2
11*	F**	44	MCA, Superior branch	Anterior Circulation	0
12	M	33	Normal	Anterior Circulation	0
13	M	30	Superior Cerebellar Artery	Posterior Circulation	1
14	F	42	Normal	Anterior Circulation	0
15	F	36	Normal	Anterior Circulation	1
16	F**	20	Normal	Anterior Circulation	0
17*	F**	35	Normal	Anterior Circulation	0
18	F**	35	PCA	Posterior Circulation	2
19*	M	35	Normal	Anterior Circulation	1
20	F	21	Normal	Anterior Circulation	2
21	F	40	MCA, Superior branch	Anterior Circulation	2
22	M	40	Normal	Anterior Circulation	2
23	F	50	MCA, Superior branch	Anterior Circulation	1
24	M	52	Superior Cerebellar Artery	Posterior Circulation	2
25	F	41	Normal	Posterior Circulation	1
26	M	19	Normal	Ophthalmoplegic Migraine	2
27	F	42	Normal	Anterior Circulation	2
28	F	42	Normal	Posterior Circulation	2
29	F	27	Normal	Posterior Circulation	0
30	F	39	Superior Cerebellar Artery	Posterior Circulation	2
31	M	44	Normal	Posterior Circulation	0
32	M	30	Normal	Anterior Circulation	0

* Recurrence of migraine induced stroke

** Recent Oral contraceptive consumption

PICA: Posterior Inferior Cerebellar Artery PCA: Posterior Cerebral Artery MCA: Middle Cerebral Artery

## Discussion

In evaluation of Framingham cohort, the age adjusted hazard ratios in women with active migraine with aura was 2.15 for ischemic stroke and women who reported active migraine without aura did not have increased risk of any vascular event.^12^

The migraine-stroke association is mostly apparent for young women with migraine with aura. This association is weaker in older age groups, which may be due to the fact that cardiovascular risk factors are more prominent with increasing age.^13^ The mean age of 37.2 years of our MIS patients is significantly lower than mean age of our whole ischemic stroke patients and MIS constitutes an important etiology of stroke in Iranian young adults.^14^ Only 32% of our MIS patients had hypodense area of brain infarction in their MRI corresponding to the neurological aura because we had a 0.5 Tesla generation MRI in our hospital which could easily lose detection of MIS. Neuroimaging demonstrates the posterior circulation as being most vulnerable in some studies, although the reason for this distribution is unclear.^15^ A preferential brainstem localization of ischemic stroke was found in a case-control series of 96 stroke patients with a life time history of migraine in Italy.^16^ Another study has shown that MIS are most frequent in the PCA territory.^16^ However, 62.5% of our MIS series had anterior circulation stroke based on the neurologic examination. Oral contraceptive consumption was found in one-third of our MIS female series. Users of oral contraceptives had eightfold increase in the risk of stroke compared with those not using these agents.^17^In 2004, The WHO stated in its medical eligibility criteria for contraceptive use that women suffering from migraine with aura, at any age, should never use oral contraceptives.^17^ Recurrence of MIS has been occurred in 12.5% of our MIS series. A third of MIS patients in a survey had recurrent events.^18^ Mean disability score of our MIS series was significantly lower than other group of stroke patients. The reason of better prognosis of MIS is unknown, but it could be due to less pathologic damage of MIS in brain parenchyma.

## Conclusion

We can decide quickly in emergency room to distinguish patient derived a benefit from invasive strategies using TIMI score. Also, TIMI risk score can be an excellent predictor to determine the extension of CAD in patients with STEMI. As a result, we should determine TIMI for any patient enters the emergency room and this score should be recorded in discharge letters.
